# Increase in gut permeability and oxidized ldl is associated with post-acute sequelae of SARS-CoV-2

**DOI:** 10.3389/fimmu.2023.1182544

**Published:** 2023-05-12

**Authors:** Christian Mouchati, Jared C. Durieux, Sokratis N. Zisis, Danielle Labbato, Michael A. Rodgers, Kate Ailstock, Brian L. Reinert, Nicholas T. Funderburg, Grace A. McComsey

**Affiliations:** ^1^ School of Medicine, Case Western Reserve University, Cleveland, OH, United States; ^2^ Center for Clinical Research, University Hospitals Cleveland Medical Center, Cleveland, OH, United States; ^3^ Division of Medical Laboratory Science, School of Health and Rehabilitation Sciences, The Ohio State University, Columbus, OH, United States

**Keywords:** COVID-19, PASC, inflammation, gut permeability, zonulin, oxidized LDL (Ox-LDL)

## Abstract

**Background:**

Post-acute sequelae of SARS-CoV-2 (PASC) is marked by persistent or newly developing symptoms beyond 4 weeks of infection. Investigating gut integrity, oxidized lipids and inflammatory markers is important for understanding PASC pathogenesis.

**Methods:**

A cross-sectional study including COVID+ with PASC, COVID+ without PASC, and COVID-negative (COVID-) participants. We measured plasma markers by enzyme-linked immunosorbent assay to assess intestinal permeability (ZONULIN), microbial translocation (lipopolysaccharide-binding protein or LBP), systemic inflammation (high-sensitivity C-reactive protein or hs-CRP), and oxidized low-density lipoprotein (Ox-LDL).

**Results:**

415 participants were enrolled in this study; 37.83% (n=157) had prior COVID diagnosis and among COVID+, 54% (n=85) had PASC. The median zonulin among COVID- was 3.37 (IQR: 2.13, 4.91) mg/mL, 3.43 (IQR: 1.65, 5.25) mg/mL among COVID+ no PASC, and highest [4.76 (IQR: 3.2, 7.35) mg/mL] among COVID+ PASC+ (p<.0001). The median ox-LDL among COVID- was 47.02 (IQR: 35.52, 62.77) U/L, 57.24 (IQR: 40.7, 75.37) U/L among COVID+ No PASC, and the highest [76.75 (IQR: 59.95, 103.28) U/L] among COVID+ PASC+ (p<.0001). COVID+ PASC+ was positively associated with zonulin (p=0.0002) and ox-LDL (p<.0001), and COVID- was negatively associated with ox-LDL (p=0.01), compared to COVID+ No PASC. Every unit increase in zonulin was associated with 44% higher predicted odds of having PASC [aOR: 1.44 (95%CI: 1.1, 1.9)] and every one-unit increase in ox-LDL was associated with more than four-fold increased odds of having PASC [aOR: 2.44 (95%CI: 1.67, 3.55)].

**Conclusions:**

PASC is associated with increased gut permeability and oxidized lipids. Further studies are needed to clarify whether these relationships are causal which could lead to targeted therapeutics.

## Introduction

The ongoing Coronavirus Disease 19 (COVID-19) pandemic dramatically changed the life of billions of people around the globe, leaving a growing number of fatalities and survivors at an unprecedented rate. The continuation of COVID-19 symptoms or the development of new symptoms, lasting more than 2 months after 4 weeks from the initial SARS-CoV-2 infection is a recognized condition referred to as long COVID or post-acute sequelae of SARS-CoV-2 (PASC) (1, 2).

It is expected that 5 to 30% of COVID survivors suffer from post-COVID symptoms, with a wide estimation interval due to the difficulty of assessment of the broad range of symptoms, the difference in the initial COVID severity, and the way PASC symptoms are assessed (self-reported *Vs*. electronic health record database (2). There are ongoing efforts to untangle the complexity of PASC in order to better understand its risk factors, pathophysiology, and possible laboratory markers to guide the development of new therapeutic plans (3).

One of the proposed theories to explain the development of PASC is the persistence of chronic inflammation after the resolution of the initial infection (4). Microbial translocation triggers inflammation by releasing microbiota or their byproducts from the gut or lung into the systemic circulation (5). Microbial translocation was also deemed responsible for organ injury and neurotoxicity by triggering metabolic dysregulations (6, 7). Intestinal microorganisms, fragments, and metabolites crossing the mucosal barrier trigger the lung inflammatory response (8). The angiotensin-converting enzyme 2 (ACE2) receptor used by SARS-CoV-2, is highly expressed in the respiratory tract and intestines, which suggests that the virus can cause long-term tissue injury and post-acute sequelae of SARS-CoV-2 infection (PASC) by compromising large mucosal surfaces (9). The continued presence of these changes can impact the ecological balance of the gut microbiome, which may play a role in the development of PASC (10, 11). Similarly, fungal translocation, another explored pathway linked to PASC, showed that an increase in tight junction permeability, measured by zonulin, and plasma β-glucan, was associated with high levels of proinflammatory cytokines in PASC patients (12).

Small studies in acute COVID-19 have measured altered levels of a biomarker for gut permeability, zonulin, in acute COVID-19; for example, a study comparing 30 hospitalized COVID patients to 35 controls found that serum zonulin levels were lower in the COVID patients (13). Another study found higher zonulin levels in patients who succumbed to severe COVID-19 (6).

Oxidized low-density lipoprotein ox-LDL, a major driver of atherosclerosis, was also blamed for driving chronic inflammation in long COVID (14). When stimulated with ox-LDL, in other inflammatory states, like atherosclerosis, macrophages responded with an increased expression of TNFα (tumor necrosis factor-alpha) and IL-6 (interleukin 6) genes (15). This present analysis is the first large study to measure zonulin and ox-LDL levels in the outpatient setting in PASC patients compared to patients with apparent complete recovery from COVID and a group of COVID-negative controls.

Our study aimed to first compare markers of gut integrity, inflammation and oxidized LDL among COVID negative patients (COVID-), COVID-positive without PASC (COVID+ No PASC), and COVID-positive with PASC (COVID+ PASC+) and second, to examine the relationships among these different markers.

## Methods

### Study design and population

This a cross-sectional study assessing inflammatory and gut integrity markers in adults 18 years or older with a confirmed COVID-19 diagnosis, either with PASC symptoms or not, compared to COVID-negative control participants. Participants were prospectively enrolled at University Hospitals Cleveland Medical Center (UHCMC), Ohio, USA. Blood was collected for measurements of selected inflammatory and gut integrity markers and oxidized LDL. The COVID+ group was subdivided into two groups, COVID+ PASC+ and COVID No PASC, based on the presence or absence of PASC symptoms obtained by participants/self-report.

A COVID-negative control group included participants from a historical, pre-COVID-19 pandemic cohort or participants with a negative SARS-CoV-2 antibody test and no prior history of COVID since January 2020. A written signed consent was obtained from all participants included; the study was approved by the Institutional Review Board (IRB) of University Hospitals Cleveland Medical Center. Participants presented fasting during all visits conducted.

### Study assessments

#### Demographics and vitals

Participants were interviewed about the key demographic information age, sex, racial group, and smoking history. We collected vital signs, including height, weight, and blood pressure. After filling out a detailed symptoms questionnaire, participants were assigned to the PASC group if they had at least two new or persistent symptoms after 1 month from the initial illness, with symptoms lasting at least 2 months (2).

#### Measurements of biomarkers

The blood collected was stored at −80°C and batched until processing without a prior thaw. Then using enzyme-linked immunosorbent assay (ELISA), we measured the following inflammatory markers: soluble tumor necrosis factor receptors I (sTNFR-I), High sensitivity C reactive protein (hs-CRP), interleukin 6 (IL-6) (R&D systems, Minneapolis, Minnesota, USA), and D-dimer (Diagnostica Stago, USA). We also measured by ELISA oxidized low-density lipoprotein (Ox-LDL) (Uppsala, Mercodia, Sweden) and two gut markers; lipopolysaccharide-binding protein (LBP) (Hycult Biotech Inc. Pennsylvania, USA), a marker of microbial translocation, and zonulin (Promocell, Germany), a marker of gut permeability. All measurements were done at Dr. Funderburg’s laboratory at Ohio State University, Columbus, Ohio.

### Statistical methods

**Figure 1:**
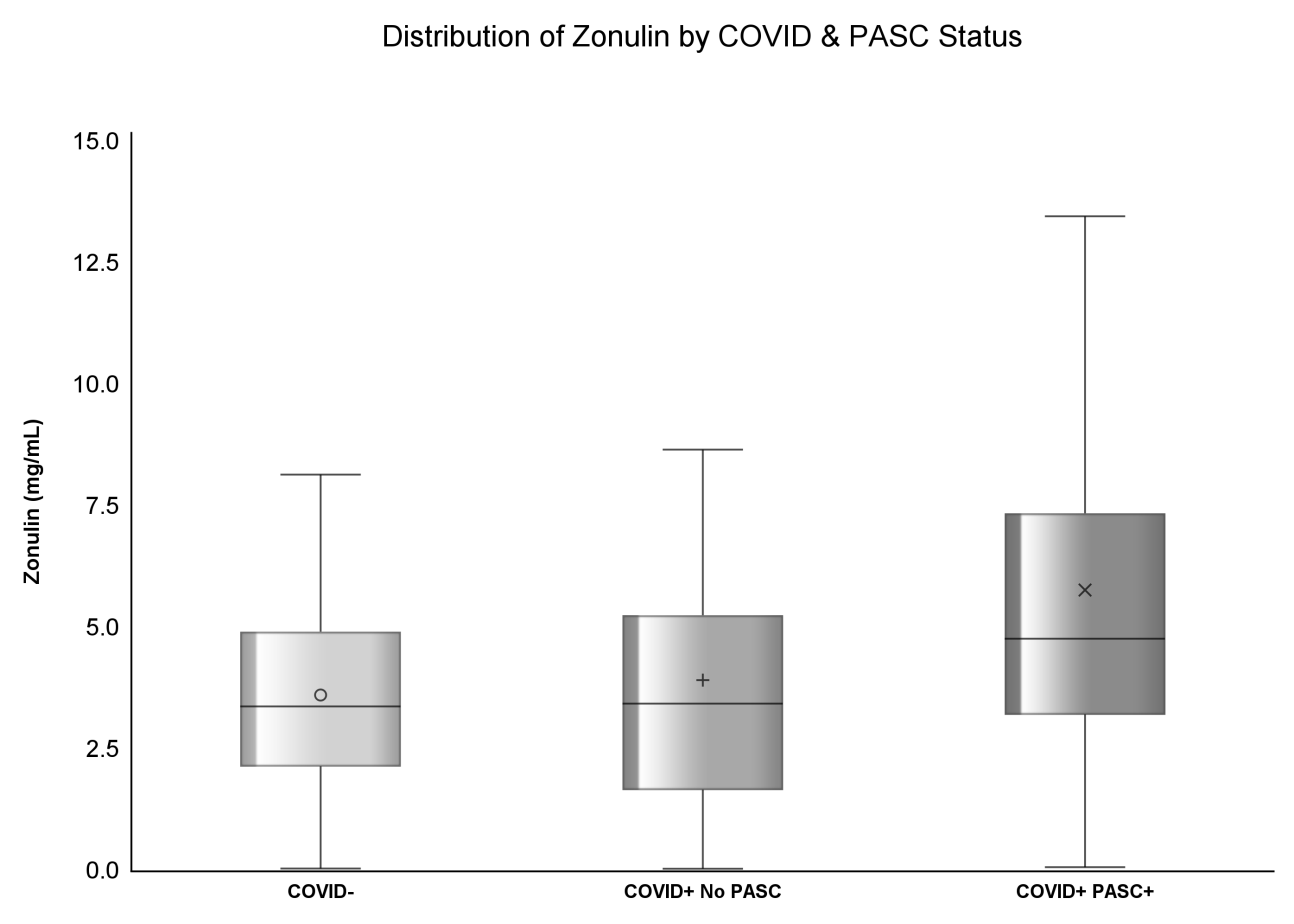
Distribution of Zonulin by COVID & PASC Status.

**Figure 2:**
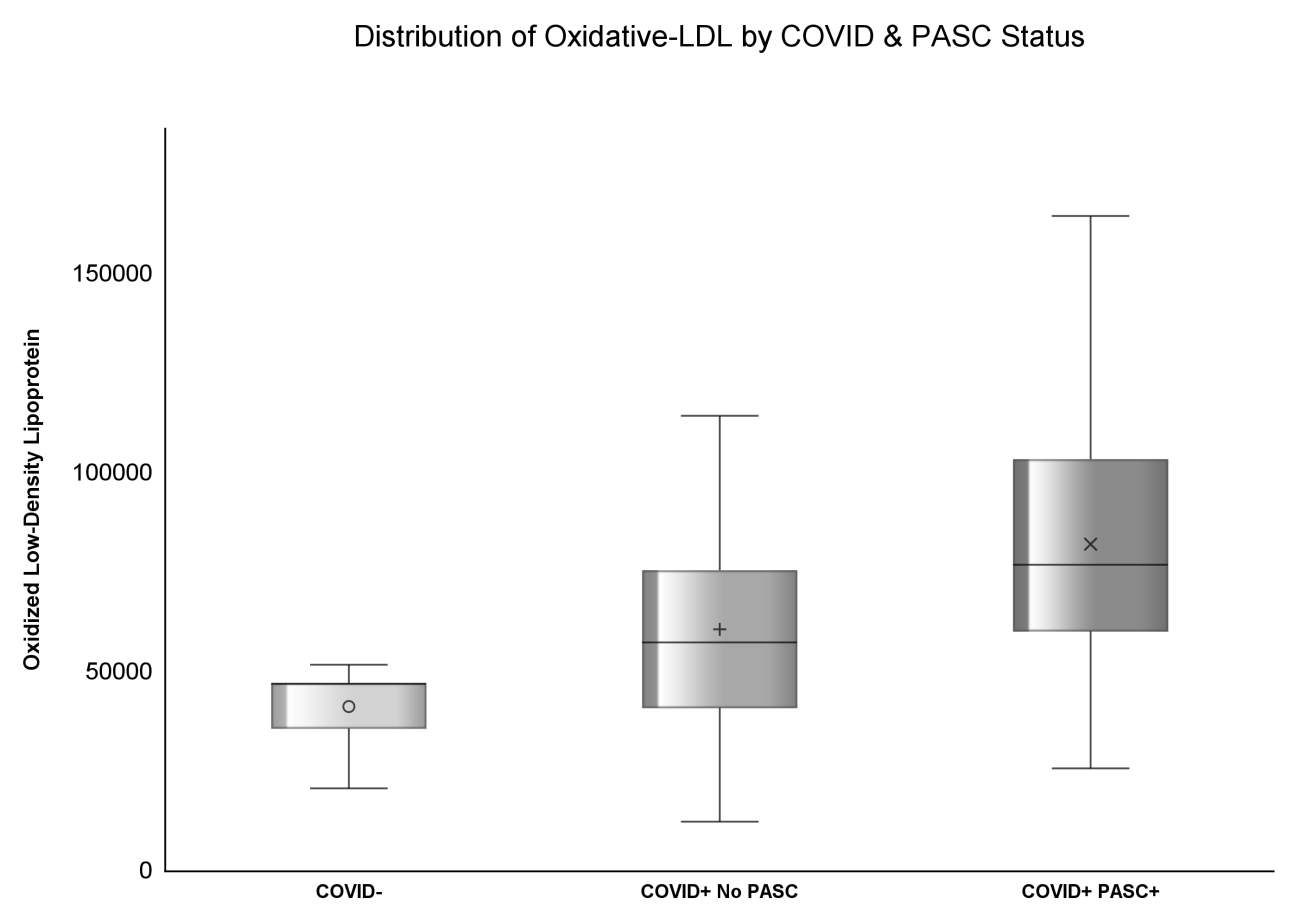
Distribution of Ox-LDL by COVID & PASC Status.

**Figure 3:**
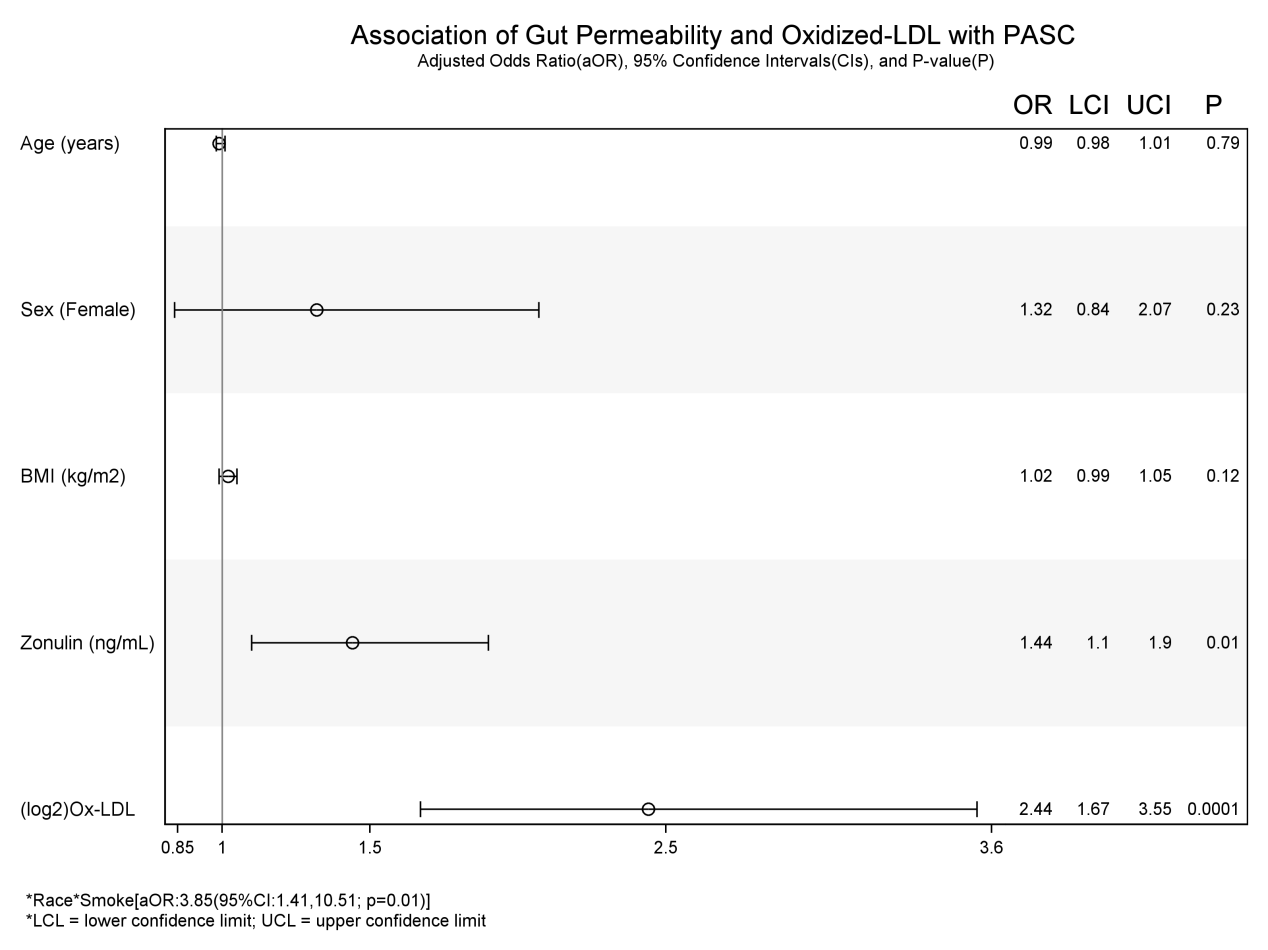
Association of Gut permeability and Inflammation with COVID+ PASC+. * interaction between the variables.

Characteristics of study participants were described using mean ± standard deviation or median and interquartile range (IQR) for continuous variables and frequency and percentage for categorical variables (**Table 1**). Differences in characteristics were assessed using Kruskal-Wallis or chi-square, or Fisher’s Exact. Generalized linear mixed models were used to assess the association between markers of gut integrity and inflammation (**Table 2**; **Figures 1**, **2**). Cumulative logit models were used to estimate the odds of PASC+ (**Table 2**) and adjusted for age, sex, race and smoking status interaction, BMI, zonulin, and inflammation marker (**Figure 3**). The proportional odds assumption was assessed using the Score test. Log transformations were used to reduce error variance and all analyses were conducted using SAS 9.4 (SAS Inc., Cary, N.C., USA). P-values less than alpha <.05 were considered statistically significant.

**Table 1:** Characteristics of participants by COVID and PASC Status.

	COVID- (n=258)	COVID+		p-value
		No PASC (n=72)	PASC+ (n=85)	
	n (%) or median (IQR) / mean ± std			
Age (years)	43.68 ± 13.69	44.85 ± 13.31	47.81 ± 13.49	0.05
Female Sex	101 (24.34)	35 (8.43)	50 (12.05)	**0.01**
Non-white Race*	108 (26.02)	25 (6.02)	26 (6.27)	0.14
BMI (kg/m2)	27.91 ± 6.05	30.68 ± 9.49	31.82 ± 8.63	**0.0002**
Current Smoker	158 (38.35)	19 (4.61)	9 (2.18)	**<.0001**
Median number of days from infection	- -	292 (IQR: 172, 518)	229 (IQR: 147, 478)	0.22
Comorbidities				
Hypertension	27 (10.47)	14 (19.44)	23 (27.06)	**0.001**
Diabetes	5 (1.94)	5 (6.94)	9 (10.59)	**0.0002**
HIV infection	98 (37.98)	22 (30.56)	22 (25.88)	0.1
Medications				
Statin	5 (1.94)	11 (14.1)	10 (9.17)	0.28
Ox-LDL (U/L)	47.02 (35.52, 62.77)	57.24 (40.7, 75.37)	76.75 (59.95, 103.28)	**<.0001**
IL-6 (pg/ml)	2.65 (1.62, 4.25)	2.21 (1.44, 3.63)	2.46 (1.77, 4.01)	0.25
D-dimer (ng/mL)	423.7 (256.02, 705.09)	429.96 (243.58, 677.41)	451.57 (266.36, 626.74)	0.93
hs-CRP (ng/ml)	2720.07 (941.82, 7205.25)	2764.87 (1188.0, 7241.84)	3182.65 (1423.9, 8931.79)	0.35
sTNF-RI (pg/ml)	1103.11 (887.11, 1375.03)	966.99 (755.06, 1331.06)	1099.98 (886.19, 1316.1)	0.08
Zonulin (mg/mL)	3.37 (2.13, 4.91)	3.43 (1.65, 5.25)	4.76 (3.2, 7.35)	**<.0001**
LBP (ng/mL)	16657.37 (11899.67, 21686.01)	14441.98 (9919.42, 23111.83)	16438.56 (10640.08, 22563.73)	0.66

*Includes African American, Asian, Hispanic, and Other Numbers in Bold indicates statistical significance (P < 0.05).

**Table 2:** Independent associations with markers of gut integrity and inflammation.

	Zonulin		Hs-CRP		Ox-LDL	
	Estimate	p-value	Estimate	p-value	Estimate	p-value
COVID & PASC status						
COVID+ PASC+ *vs*. COVID+ No PASC	0.44	**0.0002**	0.16	0.47	0.32	**<.0001**
COVID- *vs*. COVID+ No PASC	0.03	0.77	-0.06	0.73	-0.15	**0.01**
Female *vs*. Male Sex	0.19	**0.02**	0.01	0.93	0.09	**0.03**
Age	0.01	**0.03**	0.005	0.62	0.01	**0.001**
non-White *vs*. White Race	0.06	0.52	0.18	0.21	-0.09	**0.04**
BMI	0.01	0.05	0.05	**<.0001**	0.01	0.07
Current Smoker	0.3	**0.001**	0.26	0.09	-0.19	**<.0001**
Zonulin	- -	- -	0.001	**<.0001**	0.001	**0.002**
LBP	- -	- -	0.29	**<.0001**	-0.21	0.09

Numbers in Bold indicates statistical significance (P < 0.05).

## Results

### Baseline characteristics

415 participants were enrolled in this study. 62.17% (n=258) were COVID-, 20.48% (n=85) were COVID+ PASC+, and the median number of days at follow-up was 292 (IQR: 172, 518) for COVID+ No PASC and 229 (IQR: 147, 478) for COVID+ PASC+. COVID- participants were younger [COVID- (43.68±13.69 yrs.) *vs*. COVID+ no PASC (44.85±13.1 yrs.) *vs*. COVID+ PASC+ (47.81±13.49 yrs.); p=0.04], had lower BMI [COVID- (27.91±6.05 kg/m^2^) *vs*. COVID+ no PASC (30.68±9.49 kg/m^2^) *vs*. COVID+ PASC+ (31.82±8.63 kg/m^2^); p=0.0002], had the largest proportion of female sex [COVID- (24.34%) *vs*. COVID+ no PASC (8.43%) *vs*. COVID+ PASC+ (12.05%); p=0.01], and the largest proportion of current smokers [COVID- (38.35%) *vs*. COVID+ no PASC (4.61%) *vs*. COVID+ PASC+ (2.18%); p<.0001] as seen in **Table 1**. Comorbidities included HIV, hypertension and diabetes and as shown in **Table 1**, the proportions of HIV were similar across the groups.

### Gut and inflammatory markers

The median concentration of zonulin among COVID- was 3.37 (IQR: 2.13, 4.91) mg/mL, 3.43 (IQR: 1.65, 5.25) mg/mL among COVID+ No PASC, and highest [4.76 (IQR: 3.2, 7.35) mg/mL] among COVID+ PASC+ (p<.0001). Similarly, the median concentration of ox-LDL among COVID- was 47.02 (IQR: 35.52, 62.77) U/L, 57.24 (IQR: 40.7, 75.37) U/L among COVID+ No PASC, and the highest [76.75 (IQR: 59.95, 103.28) U/L] among COVID+ PASC+ (p<.0001) illustrated by **Figures 1**, **2**. Although there was a numerical variation in measured inflammatory markers, there was insufficient evidence (p>.05) to suggest any differences in IL-6, D-dimer, hs-CRP, sTNF-RI, or LBP among the three groups.

### Independent associations with markers of gut integrity and inflammation

As seen in **Table 2**, compared to COVID+ No PASC, COVID+ PASC+ was positively associated with zonulin (p=0.0002) and ox-LDL (p<.0001), and COVID- was negatively associated with ox-LDL (p=0.01). Compared to male sex, female sex was positively associated with zonulin (p=0.02) and ox-LDL (p=0.03). BMI was positively associated with hs-CRP (p<.0001), and being a current smoker was positively associated with zonulin (p=0.001) and negatively associated with ox-LDL (p<.0001). Zonulin was positively associated with hs-CRP (p<.0001) and ox-LDL (p=0.002), and LBP was positively associated with hs-CRP (p<.0001).

### Associations with COVID and PASC status

In unadjusted models (**Table 3**), age [uOR: 1.02 (95%CI: 1.002, 1.03)], female sex [uOR: 1.88 (95%CI: 1.28, 2.78)], BMI [uOR: 1.06 (95%CI: 1.03, 1.09)], ox-LDL [uOR: 6.38 (95%CI: 3.91, 10.41)], and zonulin [uOR: 1.48 (95%CI: 1.14, 1.91)] were associated with the increased odds of COVID+ PASC+. Non-white race, [uOR: 0.67 (95%CI: 0.44, 0.99)], being a current smoker [uOR: 0.13 (95%CI: 0.08, 0.22)], were less likely COVID+ PASC+. Adjusting for age, sex, the interaction between race and smoking status, BMI, zonulin (p=0.01) and ox-LDL (p=0.0001) remained associated with the increased odds of COVID+ PASC+ (**Figure 3**). Every unit increase in zonulin was associated with 44% higher predicted odds of having PASC [aOR: 1.44 (95%CI: 1.1, 1.9)] and every one-unit increase in ox-LDL was associated with more than four-fold increased odds of having PASC [aOR: 2.44 (95%CI: 1.67, 3.55)].

**Table 3:** Independent associations with COVID+ PASC+.

	uOR (95% CI)*	p-value
Age	1.02 (1.002, 1.03)	**0.02**
Female *vs*. Male Sex	1.88 (1.28, 2.78)	**0.001**
non-White *vs*. White Race	0.67 (0.44, 0.99)	**0.04**
BMI	1.06 (1.03, 1.09)	**<.0001**
Current Smoker	0.13 (0.08, 0.22)	**<.0001**
IL-6	0.84 (0.65, 1.09)	0.2
hs-CRP	1.09 (0.95, 1.26)	0.23
D-dimer	0.86 (0.68, 1.07)	0.18
Ox-LDL	6.38 (3.91, 10.41)	**<.0001**
Zonulin	1.48 (1.14, 1.91)	**0.003**
LBP	0.8 (0.61, 1.06)	0.12

*uOR=Unadjusted Odds Ratio and 95% Confidence Intervals.

Numbers in Bold indicates statistical significance (P < 0.05).

## Discussion

Post-acute sequelae of SARS-CoV-2 infection is a well-established condition considered a public health priority. PASC incidence is increasing, but its pathophysiology is still poorly understood; the research community is tirelessly trying to narrow the knowledge gap, and any contribution could be a valuable piece of the puzzle.

Recent studies have assessed the association between PASC and inflammation. The disruption of the gut-lung axis, a known severity marker in other respiratory diseases, could also be a contributing factor (16). SARS-CoV-2 can affect the gastrointestinal tract directly or indirectly, leading to disruptions in gut barrier integrity, allowing gut microbes and their products to translocate across the gut epithelium and exacerbate initial systemic inflammation (17, 18). In addition, acute COVID-19 has been associated with increased plasma levels of zonulin, a marker of tight junction permeability, leading to microbial translocation and increased inflammation (19, 20). Our study reports that the mean plasma concentration of zonulin was the highest among COVID+ PASC+ compared to levels in both COVID+ No PASC or COVID- participants, even after adjusting for race, smoking status, and BMI. Moreover, an increase in zonulin is associated with increases in hs-CRP and Ox-LDL; thus, we suspect that zonulin may be a key driver of inflammation in PASC. Zonulin was associated with increased COVID severity in adults and children with multisystem inflammatory syndrome (21, 22), and the association of zonulin with neurological symptoms in hospitalized COVID-19 patients was described (23). To the best of our knowledge, we are the first to assess zonulin along with other gut markers, inflammatory markers and oxidized LDL in a large outpatient group in the post-COVID setting.

Oxidized LDL may be another potential driver of inflammation in PASC as it can activate the inflammasome through Toll-like receptor 4 and CD36 binding (24); we report here that the mean ox-LDL concentration was the lowest among COVID- and the highest among COVID+PASC+, even after adjusting for race, smoking status, and BMI. Ox-LDL was associated with hyperinflammation in acute COVID-19 (25, 26); at the time of this writing, this is the first large cohort to measure the levels of ox-LDL in PASC patients.

Furthermore, LBP was positively associated with hs-CRP, despite the fact that the following biomarkers showed no statistical significance between the different groups: IL-6, D-dimer, hs-CRP, sTNF-RI, and LBP. The discrepancy between LBP and hs-CRP findings may relate to the assay variability of some of the inflammation markers, such as hs-CRP. Another possibility would be that elevated hs-CRP, and other inflammation markers may be driven by multiple factors, gut dysfunction being one of them. Our findings are in line with studies conducted in Brazil and Spain that found no differences in IL-6 or D-dimer between PASC and post-COVID without PASC (27, 28). In contrast, one study reported that hs-CRP was correlated with post-acute COVID-19 (29). The discrepancies in biomarkers results observed between the studies could be due to the sample collection’s different timing or different comorbidities or PASC symptomatology in the studied population. A recent meta-analysis revealed that several studies found elevated inflammation markers in PASC patients, although there was variability among markers used and variability in results by study (30). Of note, none of these studies had measured oxidized LDL or gut markers along with inflammatory markers as we have performed here.

We acknowledge that our study has several limitations. The cross-sectional nature of this study cannot establish causation between PASC+ status and inflammatory and gut markers.

Despite adjusting for age, sex, race, BMI, and smoking status, the biomarkers could have been affected by other confounders. Also, the data on previous hospitalization was not available.

In conclusion, PASC is associated with increased ox-LDL and increased gut permeability, which in turn is associated with oxidized LDL and hs-CRP. In addition, the microbial translocation marker LBP was also positively associated with hs-CRP. Further research is needed for a deeper understanding of the causative role of gut dysfunction and oxidative stress in PASC which could provide important therapeutic targets.

## Data availability statement

The raw data supporting the conclusions of this article will be made available by the authors, without undue reservation.

## Ethics statement

The studies involving human participants were reviewed and approved by the Institutional Review Board (IRB) of University Hospitals Cleveland Medical Center. The patients/participants provided their written informed consent to participate in this study.

## Author contributions

CM, JD, SZ, and GM contributed to the study concept and design. All authors contributed to the acquisition of data. JD and GM contributed to the analysis and interpretation of data. CM, JD, SZ, and GM drafted the manuscript. All authors contributed to the critical revision of the manuscript for important intellectual content. JD contributed to statistical analysis. GM obtained funding. CM, SZ, JD, DL, MR, KA, BR, and NF contributed to administrative, technical, or material support. GM supervised the study. All authors contributed to the article and approved the submitted version.
